# The ATP binding cassette transporter, ABCG1, localizes to cortical actin filaments

**DOI:** 10.1038/srep42025

**Published:** 2017-02-06

**Authors:** Elvis Pandzic, Ingrid C. Gelissen, Renee Whan, Philip J. Barter, Dmitri Sviridov, Katharina Gaus, Kerry-Anne Rye, Blake J. Cochran

**Affiliations:** 1 Biomedical Imaging Facility, Mark Wainwright Analytical Centre, UNSW Australia, Sydney, Australia; 2 EMBL Australia Node in Single Molecule Science, School of Medical Sciences, UNSW Australia, Sydney, Australia; 3 Faculty of Pharmacy, University of Sydney, Sydney, Australia; 4 School of Medical Sciences, Faculty of Medicine, UNSW Australia, Sydney, Australia; 5 Faculty of Medicine, University of Sydney, Australia; 6 Baker IDI Heart and Diabetes Institute, Melbourne, Australia; 7 ARC Centre of Excellence in Advanced Molecular Imaging, UNSW Australia, Sydney, Australia

## Abstract

The ATP-binding cassette sub-family G member 1 (ABCG1) exports cellular cholesterol to high-density lipoproteins (HDL). However, a number of recent studies have suggested ABCG1 is predominantly localised to intracellular membranes. In this study, we found that ABCG1 was organized into two distinct cellular pools: one at the plasma membrane and the other associated with the endoplasmic reticulum (ER). The plasma membrane fraction was organized into filamentous structures that were associated with cortical actin filaments. Inhibition of actin polymerization resulted in complete disruption of ABCG1 filaments. Cholesterol loading of the cells increased the formation of the filamentous ABCG1, the proximity of filamentous ABCG1 to actin filaments and the diffusion rate of membrane associated ABCG1. Our findings suggest that the actin cytoskeleton plays a critical role in the plasma membrane localization of ABCG1.

The ATP-binding cassette (ABC) sub-family G member 1 (ABCG1) is a transmembrane half transporter that exports cellular lipids to extracellular acceptors^1^,^2^. The primary function of ABCG1 is to efflux cholesterol to spherical high-density lipoproteins (HDL). ABCG1 also effluxes cholesterol to low-density lipoproteins (LDL), liposomes and cyclodextrin^3^,^4^,^5^ and it exports sphingomyelin, phosphatidylcholine and oxysterols to HDL and albumin^6^,^7^. As ABCG1 is a half transporter^8^, dimerization is required for function^4^. Whilst ABCG1 is generally considered to function as a homodimer^9^, recent evidence suggests it also forms heterodimers with ABCG4^10^,^11^. There has been significant debate over the precise cellular localization and, by extension, function of ABCG1. A number of studies have reported that ABCG1 is localized and functions in lipid rafts in the plasma membrane^4^,^12^,^13^,^14^,^15^ and in endosomes that recycle to the cell surface where they efflux cholesterol^13^. However, other reports have suggested that ABCG1 is restricted to endosomes^16^ and secretory granules in pancreatic beta-cells^17^.

It has recently been suggested that ABC transporters are stabilized by association with an actin-dependent subtype of lipid rafts. This association appears to regulate transporter function^18^. A recent single molecule investigation of the dynamics of the cholesterol transporter, ABCA1, at the plasma membrane revealed that the actin cytoskeleton stabilizes transporter dimerization^19^ in a process that is a prerequisite for mediating cholesterol efflux to apolipoprotein (apo) A-I, the primary apolipoprotein of HDL. Given that ABCA1 and ABCG1 both efflux cholesterol from the cell surface to the extracellular space, and the requirement of ABCG1 dimerization for function, we hypothesized that ABCG1 can also associate with actin. Support for this hypothesis comes from Aleidi *et al*.^20^ who identified a number of cytoskeletal-associated proteins including alpha-tubulin, cytoskeleton-associated protein 5 and dynamin as ABCG1 interacting partners. In this study, we examined the spatial organization of ABCG1 in filamentous structures that co-localize with the actin cytoskeleton proximal to the plasma membrane. We employed total internal reflection fluorescence microscopy together with plasma membrane sheets to establish that a sub-population of ABCG1 is localized to cortical actin filaments.

## Results

### ABCG1 organizes into filamentous surface membrane associated structures and is enhanced by cholesterol loading

**Figure 1:**
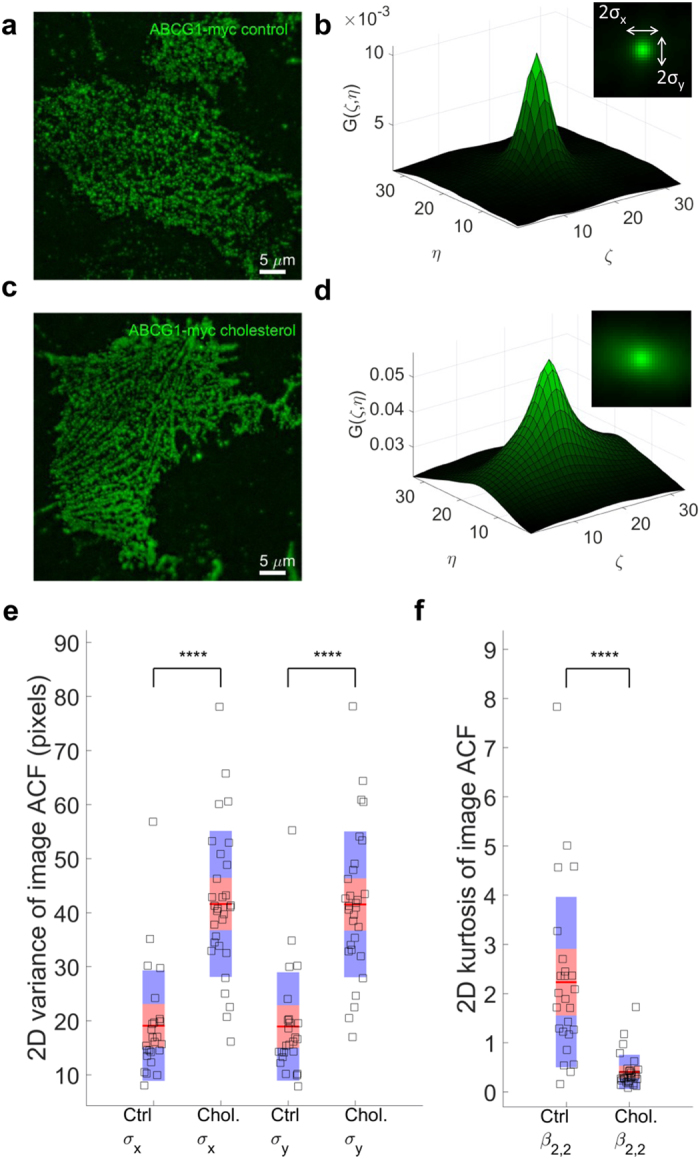
ABCG1 organized into filamentous structures at the plasma membrane. After membrane removal and imaging of the remaining membrane sheet in control (**a**) or cholesterol treated (**c**) cells, the filaments are more visible. (**b**,**d**) are examples of 2D ICS auto-correlation functions (ACF) for control and cholesterol treated cells, respectively. Insets in (**b**,**d**) display the top view of 2D ACFs, indicating the extent of the 2D variances of these distributions. The 2D variances of ACFs, shown in (**e**) for cholesterol treated (n = 30 cells) cells show much larger variances than controls (n = 25 cells), indicating that cholesterol increases the filamentation pattern and the spread of 2D ACFs. This is in agreement with much lower 2D kurtosis (peakedness) of cholesterol treatment ACFs, shown in (**f**). The mean of the data is shown as a red line. The 95% confidence intervals are represented by the pink zones and one standard deviation is represented by the blue zones ^****^P < 0.0001.

We first employed total internal reflection fluorescence (TIRF) microscopy to visualize the plasma membrane localization of ABCG1 in CHO-K1 and HeLa cells stably overexpressing human ABCG1. The presence of ABCG1 in continuous filaments was evident in intact, fixed cells (Supplementary Figs 1–4). We hypothesized that these filamentous structures were present on the plasma membrane, but were partially obscured due to co-localisation of ABCG1 sub-populations adjacent to the membrane and within the TIRF microscopy range (200 nm). In order to better visualise the filamentous structures, membrane sheets were prepared (see Methods) so that organization of ABCG1 in the basal membrane could be assessed (Fig. 1a,c). Images of membrane sheets prepared from control cells (Fig. 1a, Supplementary Fig. 1) or cells pre-treated with methyl-β-cyclodextrin/cholesterol complexes (Fig. 1c, Supplementary Fig. 2) were quantified using a combination of Image Correlation spectroscopy (ICS) and 2D image moments. When ICS is applied to the images of membrane sheets from control samples, the auto-correlation function (ACF) exhibits more a symmetric, peaked shape (Fig. 1b). The variance of the ACF values was significantly increased in the cholesterol-loaded cells (Fig. 1e), with the peaks showing wider shoulders and long range spatial lags (Fig. 1d). This is due to the long range correlations of adjacent pixel intensity, resulting in decreased kurtosis (peakedness) (Fig. 1f) and is indicative of an increase in the filamentous structure of ABCG1. Cholesterol treatment did not alter the localization of the myristoyl anchored eGFP reporter Src15-eGFP (Supplementary Fig. 5)

### Filamentous ABCG1 does not localize with ER

**Figure 2:**
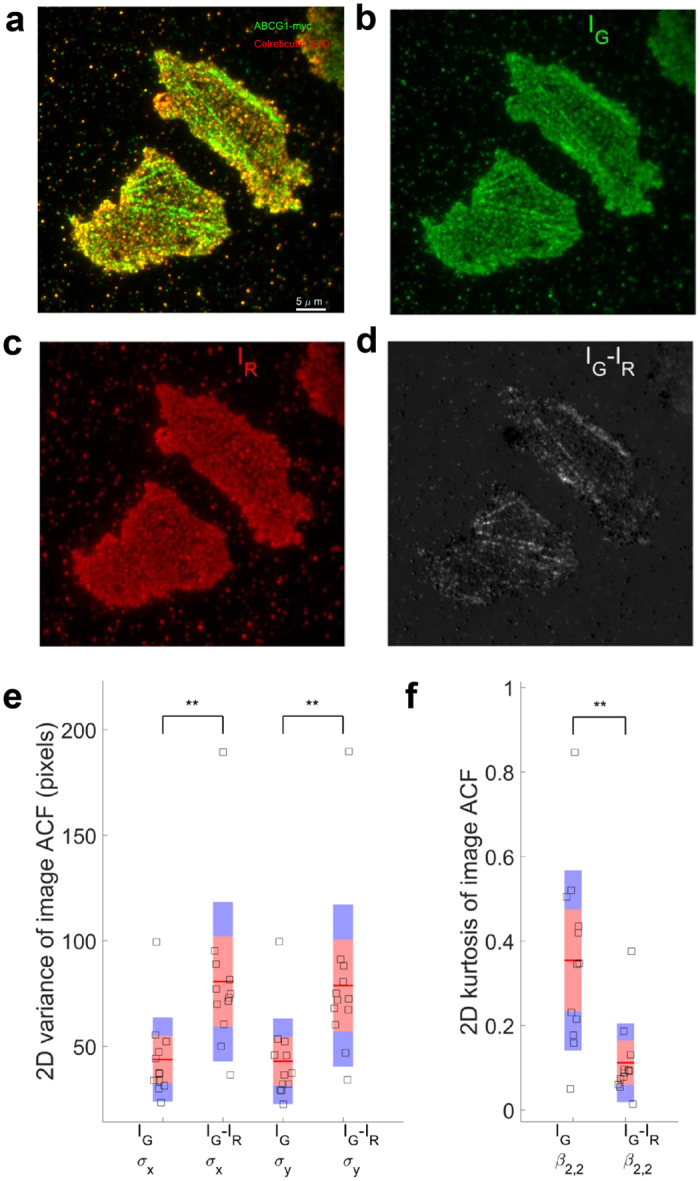
Filamentous ABCG1 does not localize with calreticulin in the ER. (**a**) Representative example of intact, untreated, permeabilized and fixed cells immunostained for ABCG1-myc (green) and the ER marker calreticulin (red). (**b**) Green (I_*G*_) and (**c**) red (I_*R*_) channels images and (**d**) the difference (I_*G*_-I_*R*_) image. (**e**) The variances of the ACF of the difference images (n = 12) are larger than that of green channel alone, indicating that ABCG1 that did not co-localize with ER tended to be filamentous in nature. (**f**) 2D Kurtosis displays lower peakedness in the difference image than in the green channel alone ^**^P < 0.01.

The crossing filamentous structures produced comb-like features that were reminiscent of an endoplasmic reticulum (ER) network adjacent to the plasma membrane. To determine if this was the case, colocalization of the ABCG1 filaments with calreticulin in the ER was evaluated by imaging intact, untreated, fixed cells expressing ABCG1 and immunostaining for ABCG1 and calreticulin. Co-localization of the ER and ABCG1 was observed as previously reported^12^ in both control and cholesterol treated cells (Fig. 2a, Supplementary Figs 6 and 7). However, multiple ABCG1 filaments did not co-localize with the ER under both control and cholesterol treated conditions. The difference image (Fig. 2d) between the normalized green (Fig. 2b) and red (Fig. 2c) channel images clearly showed filamentous ABCG1 structures were not co-localized with calreticulin, representing a separate pool of ABCG1. Quantification of ACFs of the green channel (ABCG1) and the difference image (I_*G*_-I_*R*_) was consistent with the visible differences in Fig. 2c containing ABCG1 primarily localized in filaments that were not co-localized with calreticulin (Fig. 2e,f). Importantly, no calreticulin staining was observed on membrane sheets (data not shown).

### ABCG1 localizes in the proximity of actin filaments

**Figure 3:**
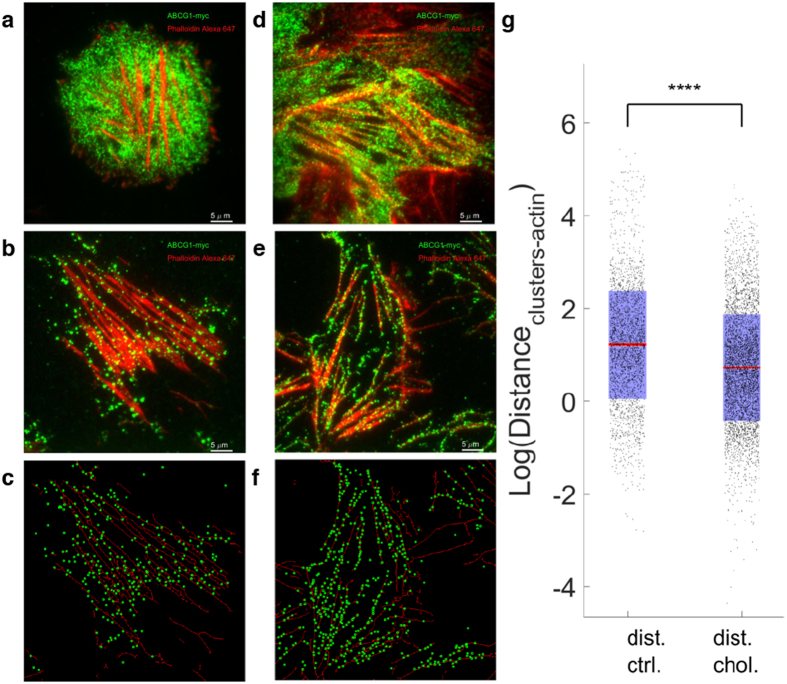
Filamentous ABCG1 co-localizes with actin filaments. Colocalization of ABCG1 (green) and actin (red) filaments under control conditions in intact, fixed cells (**a**) and cell membrane sheets (**b**) and in cholesterol treated intact, fixed cells (**d**) and cell membrane sheets (**e**). (**c,f**) Detected ABCG1 clusters (circles) superimposed on a binary skeletonization mask from actin filament image. (**g**) Quantification of distance (pixels) between ABCG1 clusters and closest actin skeleton point ^****^P < 0.0001.

The filamentous nature of the ABCG1 in the basal membrane prompted us ask if this organization could be explained by co-localization of ABCG1 with the actin cytoskeleton. Labeling of actin filaments and ABCG1 in intact, fixed, and untreated cells showed clusters of ABCG1 (green) lining actin filaments (red) (Fig. 3a, Supplementary Fig. 8). Co-localization and coupling of ABCG1 with the actin filaments in intact cells was even more pronounced in cells that were pre-incubated with cholesterol-loaded β-methyl cyclodextrin (Fig. 3d, Supplementary Fig. 9). In order to better characterize this co-localization, we prepared basal membrane sheets, which confirmed that this co-localization occurred at the cell membrane in both untreated (Fig. 3b) and cholesterol loaded cells (Fig. 3e). To quantify the impact of cholesterol loading on ABCG1-actin co-localization, actin filament images were converted into bare skeleton binary images (Supplementary Fig. 10; see Methods for details) and ABCG1 cluster positions extracted. This information (Fig. 3c,f) was used to calculate the distance between ABCG1 cluster centroids and the closest filament of the actin network (Fig. 3g). Cholesterol loaded cells show smaller average distance between ABCG1 positions and actin filaments (0.19 ± 0.18 μm) relative to control cells (0.34 ± 0.29 μm).

### Inhibiting actin polymerization disrupts filamentous ABCG1

**Figure 4:**
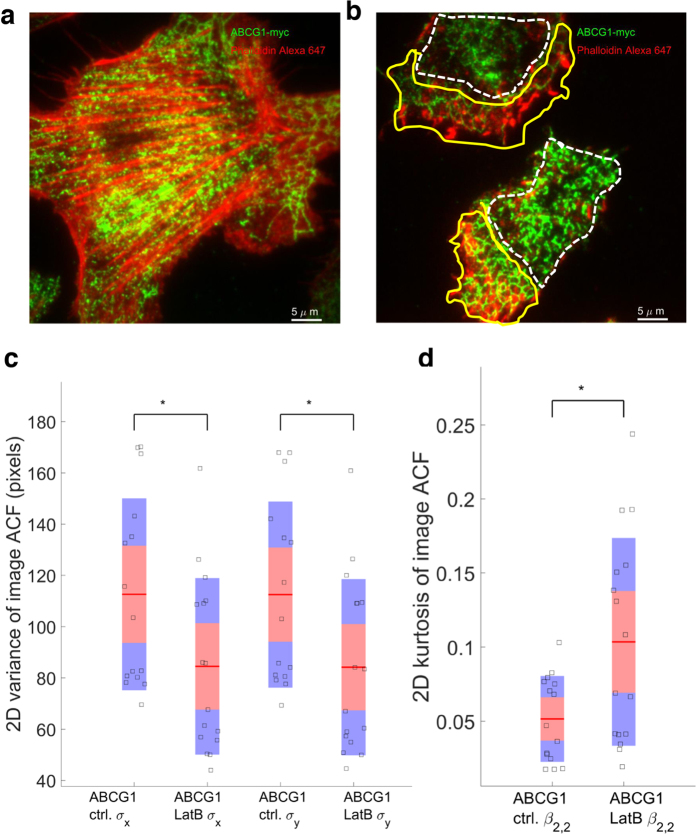
Filamentous ABCG1 is disrupted by the actin polymerization blocking agent, Latrunculin B. ABCG1 and actin localisation in control (**a**) and Latrunculin B treated cells (**b**). White dashed lines show the regions in which ABCG1 organizes in heterogeneous clusters, while yellow lines delimit areas of cell exhibiting more structured, comb-like ABCG1 organization. (**c**) The variances of the ACF of the Latrunculin B cells were lower than control cells, indicating a loss of ABCG1 and actin association. (**d**) 2D Kurtosis displays higher peakedness in the Latrunculin B image than in the control image ^*^P < 0.05.

Disruption of actin filaments in intact CHO-K1 cells with Latrunculin B resulted in loss filamentous actin and ABCG1 structures (Fig. 4a control vs 4b treated; Supplementary Fig. 11). The depolymerized actin monomers aggregated in the cellular periphery and were associated with the reorganization of ABCG1 into comb-like structures (yellow lines). Coincidentally, regions of cells enclosed by yellow lines display a higher concentration of actin relative to regions enclosed by white lines. The variance of the ACF values was significantly decreased in the Latrunculin B treated cells (Fig. 4c), resulting in increased kurtosis (peakedness) (Fig. 4d) due to loss of ABCG1 filamentous structure.

### Plasma membrane ABCG1 colocalizes with Lck10

**Figure 5:**
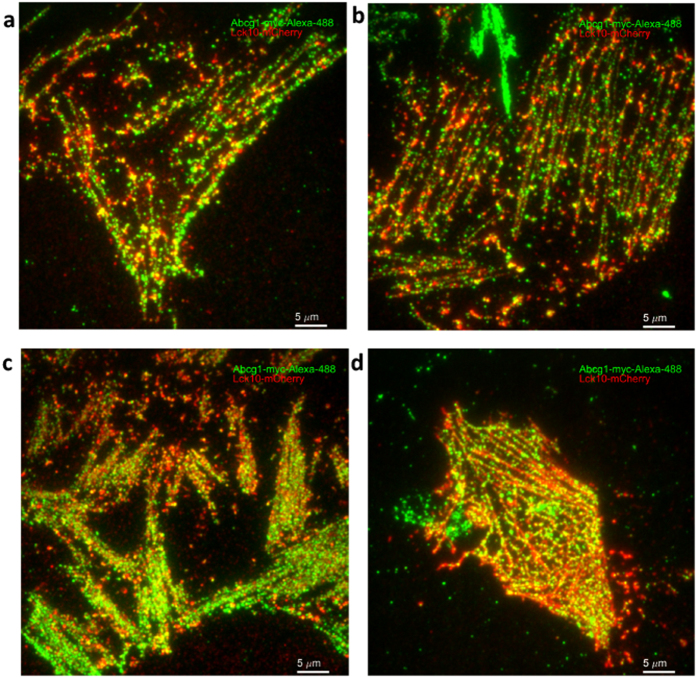
Filamentous ABCG1 co-localizes with Lck10. ABCG1 (green) and Lck10 (red) localization in control (**a,b**) and cholesterol treated (**c,d**) CHO-K1 cell membrane sheets.

To further understand the localization of filamentous ABCG1, an inner membrane leaflet peptide, Lck10-mCherry, was expressed in CHO-K1 cells. Lck10 was previously shown to organize along actin filaments in cholesterol rich plasma membrane domains^21^. Filamentous organization of both Lck10 and ABCG1 was observed in cell membrane sheets under control and cholesterol treated conditions (Fig. 5). Lck10 formed continuous filaments similar to that previously observed for actin, with ABCG1 aligned with Lck10 and localized to punctuated clusters.

### Cholesterol increases ABCG1 diffusion

**Figure 6:**
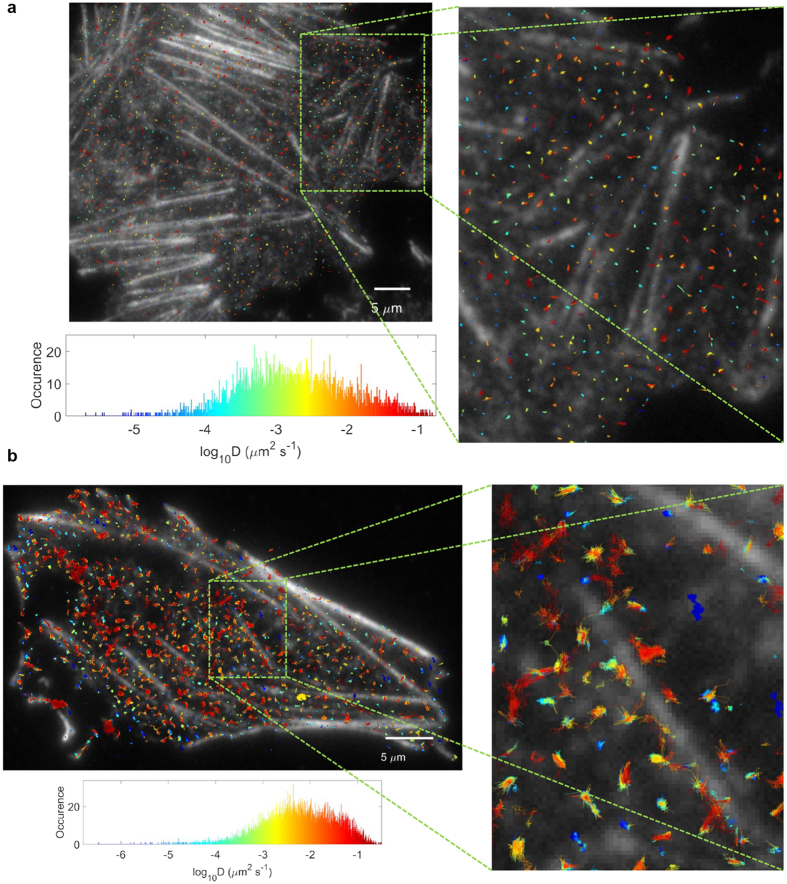
Cholesterol increases ABCG1 diffusion. (**a**) Example of control sample ABCG1 trajectories in control cells, colour coded by the magnitude of the diffusion coefficient (blue to red for increasing D, see histogram) and superimposed on the image of phalloidin-labeled actin. (**b**) Example of trajectories for ABCG1 in cholesterol-loaded CHO-K1 cells. The mean square displacement for each ABCG1 trajectory was calculated and fitted linearly for the first 5 temporal lags, in order to obtain a diffusion coefficient per trajectory. The membrane sheets were imaged at 26 °C.

In order to explore further the association of ABCG1 and actin filaments, we evaluated the mobility of ABCG1 by time series imaging of membrane sheets from non-fixed cells. Single particle tracking of ABCG1 in the control samples did not exhibit large displacements (Fig. 6a) while the cholesterol-loaded cells showed trajectories oriented along the actin filaments (Fig. 6b). The largest diffusion coefficient observed was in the order of 0.1–0.5 s, and the trajectories were generally localized to regions between actin filaments (Fig. 6b). The mean diffusion coefficient ± standard deviation for control sample was 1.7 ± 3.5 ms. The ABCG1 in the cholesterol-loaded cells showed a faster average diffusion coefficient of 4.7 ± 7.7 ms.

## Discussion

The cellular localization and, by extension, the function of ABCG1 has been a topic of significant debate. Whilst a number of investigators have reported that ABCG1 exports cholesterol to extracellular acceptors and that it is localized at the cell surface^4^,^12^,^13^,^14^, others have reported that it functions intracellularly^16^. In this study, we utilized TIRF microscopy and cell membrane sheet preparations to resolve these conflicting reports and establish unequivocally whether ABCG1 is localized at the plasma membrane and precisely which intracellular compartments with which it associates.

Our findings indicate that ABCG1 is organised in two distinct cellular pools that are located in the ER and at the plasma membrane. The presence of ABCG1 in the ER is consistent with its previously described intracellular role in the removal of excess lipid from intracellular compartments^16^. However, that study found no colocalisation between ABCG1 and the ER marker calnexin^16^. We also observed that ABCG1 that was not colocalised with the ER marker calreticulin, but that it was organised into filamentous structures. As TIRF microscopy allows for visualisation of molecules within approximately 100 nm of the plasma membrane, we prepared cell membrane sheets to confirm that these filamentous structures were localized at the plasma membrane.

Treatment of cells with cholesterol increased plasma membrane associated ABCG1 levels indicating that ABCG1 cycles between the intracellular and plasma membrane pools based on cellular cholesterol levels. This is consistent with a previous study that ascribed an exclusively intracellular role to ABCG1 in cells maintained in lipoprotein deficient serum and in the presence of a statin^16^, both of which are associated with decreased cellular cholesterol levels. In addition to regulating ABCG1 localization, cholesterol also regulates ABCG1 levels via inhibition of ubiquitination and subsequent degradation^22^.

The organization of ABCG1 into filamentous structures prompted us to investigate if plasma membrane associated ABCG1 was associated with cortical actin filaments. ABCG1 interacts with a number of actin-associated proteins^20^ and has previously been shown to localize to RhoB + vesicles^23^, which regulate endosome transport to subcortical actin stress fibres^24^. In the present study ABCG1 co-localized with actin in basal membrane sheets and its organization was severely disrupted by inhibition of actin polymerization. This strongly suggests that surface-associated ABCG1 is linked to the actin cytoskeleton. It is also consistent with reports of actin polymerization impacting on the localization of ABCA1^19^ and functionality of ABCC1^25^. Loading of cells with cholesterol significantly increased the organization of ABCG1 in the cell membrane into filamentous structures. The strong alignment of ABCG1 and Lck10 in filamentous structures on membrane sheets provides further evidence that ABCG1 is present at the cell surface and is localized in cholesterol rich, actin associated membrane regions.

In summary, this study resolves the discrepancies regarding the intracellular organization of ABCG1 and demonstrated that the actin cytoskeleton plays a critical role in the plasma membrane localization of ABCG1.

## Methods

### Cell Culture and Treatment

CHO-K1 cells stably overexpressing *C*-terminally myc-tagged ABCG1^2^,^9^ were cultured in Ham’s F12 medium supplemented with 10% (v/v) heat inactivated FBS at 37 °C in 5% CO_2_. HeLa cells stably overexpressing ABCG1-GFP^26^ were cultured in DMEM medium supplemented with 10% (v/v) heat inactivated FBS at 37 °C in 5% CO_2_. Cells were seeded in a glass-base dish (35 mm diameter with a window diameter of 12 mm, 0.15 mm-thick glass; Iwaki) and cultured for 1 d before experimenation. The cells were enriched with cholesterol by incubation at 37 °C for 30 min with methyl-β-cyclodextrin/cholesterol complexes (Sigma-Aldrich) at a final cholesterol concentration of 50 μg/mL. Actin polymerization was inhibited in by incubating the cells at 37 °C for 30 min with Latrunculin B (final concentration 10 μM).

### Cell Membrane Sheet Preparation

In order to isolate the basal membrane and associated actin filaments from CHO-K1 cells, membranes were sheared off cells adhering to the glass coverslip of an imaging dish as previously described^27^,^28^. Culture media was removed and the cells were washed with PBS, followed by osmotic swelling (1 min) with sterile water. Ripping stamps were prepared in advance, using round 18 mm coverslips that were cleaned with water, acetone, ethanol and super-glued onto a 1.5 mL Eppendorf tube. A drop of 0.01% Poly-L-Lysine (PLL, Sigma-Aldrich) was deposited on the clean side of the coverslip and incubated at room temperature for 30 min. Non-deposited PLL was rinsed off using distilled water. The water was removed from cells and the PLL coated side of the stamp was pressed onto the surface of cells. The stamp was removed along with the cell membrane. The culture dish was rinsed twice with PBS to remove remaining organelles from the exposed cellular cytosol and fixed in 10% (w/v) neutral buffered formalin for 10 min at room temperature.

### Labelling

Whole cells and cell sheets were blocked/permeabilized overnight at 4 °C in PBS containing 1% (w/v) BSA, and 0.3% (v/v) Triton X-100. The samples were washed in PBS and stained with Alexa488 conjugated myc-tag antibody (Cell Signaling), calreticulin (ER marker) antibody (Abcam) or Alexa647 conjugated phalloidin (Thermo Fisher Scientific) for 1 h at room temperature. The samples were washed and, where required, incubated with Alexa647 conjugated anti-rabbit antibody for labelling of calreticulin for 1 h. All samples were washed in PBS prior to imaging. To allow for comparison of different acquisition channels and generation of difference images, images from each channel were normalized relative to their maximum intensity, such that final intensity ranged from 0 to 1.

### Transfection

CHO-K1 cells stably overexpressing *C*-terminally myc-tagged ABCG1 were transfected with plasmid encoding Lck10-mCherry or Src15-eGFP^21^ using Lipofectamine 3000 (ThermoFisher Scientific). Protein expression was confirmed after 48 h using a fluorescence microscope.

### TIRF Imaging

Imaging was performed on an Elyra (Zeiss) microscope using TIRF illumination. Alexa488-myc labelled ABCG1, ABCG1-eGFP and Src-eGFP were imaged using 488 nm and phalloidin Alexa647 labelled actin and mCherry were imaged using 642 nm laser lines, sequentially with appropriate band-pass filters to minimize the bleed-through. A high resolution 100×, 1.46 NA Oil immersion objective lens was used for imaging, at 1.6× additional zoom, resulting in a 0.1 μm pixels size. For time series experiments, images were acquired at 32 Hz.

### ICS Analysis and Image Moments of ACF

Spatial auto-correlation function (ACF) for each image was calculated as described previously^29^. Briefly, images were Fast Fourier Transformed (FFT) spatially and an image spectrum obtained by multiplication with its complex conjugate. The inverse FFT was applied to the absolute value of the spectrum which resulted in the spatial ACF. This approach to image ACF calculation saves computational time, without affecting the final result. In order to characterize the 2D variance and peakedness of this ACF distribution, we calculated the first 4 moments using the method as described previously^30^. In summary, 2D variances along x and y axis were obtained using:


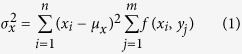



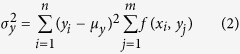


where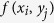 denotes the amplitudes of ACF at spatial lag 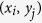 and 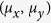 are the means of the 2D distribution, or in this case the spatial lags at which ACF peaks (i.e. 0,0). 2D Kurtosis was obtained using:


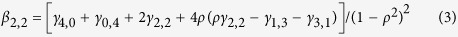


and


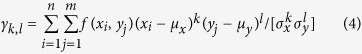


For each ACF a σ_x_, σ_y_ and β_2,2_ were calculated and summarized for a given condition.

### Actin Skeletonization and ABCG1 Feature Finding

The actin image was passed through the *A trou* wavelet filter as described previously^31^, in order to smooth the image and remove the noise. The filtered image was thresholded so that all the values above the mean intensity +10% of standard deviation of intensity distribution, were kept in the mask. The binary mask was cleaned by morphological operations using Matlab built in operation of opening, skeletonization and bridging of isolated pixels. The ABCG1 clusters detection was performed using a previously developed Matlab code^32^. The centroids of each cluster was extracted and used for further distance measurement to the actin skeleton, which was done using custom built Matlab code.

### Single Particle Tracking of ABCG1 Clusters and Mean Square Displacement Analysis

The single particle tracking was done using the DiaTrack^33^ algorithm. The image series were loaded in DiaTrack GUI and Gaussian filter of σ = 1.3 pixels applied to remove the background noise. For each cell analyzed, a contour polygon around the cell was selected to increase the speed of the detection and tracking and remove the undesired features from the extracellular background. A threshold of ∼15–25 out of 255 was sufficient to cutoff the faint and noisy features detected. After feature detection, the tracking maximum particle displacement, in pixels, was estimated using the toggle option of the GUI. After tracking, the tracks were bridged, for the gaps, using the post-processing options of the GUI. Finally, the trajectories were corrected for xy drift, using the post-processing option of drift correction using the overall movement of the center of mass of particles. The tracking results were imported in Matlab and the mean square displacements were calculated per trajectory and fitted for first five temporal lags, in order to extract the diffusion coefficient per trajectory. The trajectories were superimposed on the actin image and color coded by the magnitude of the diffusion coefficient.

### Statistical Analysis

The data points in the statistical plots show the central mark which is the mean of the data (red line). Points are visualized as a 1.96 standard error of the mean (95% confidence interval) in pink zones and 1 standard deviation in blue zones. One-way Anova of the mean was used to determine statistically significant differences between data sets. P < 0.05 was considered statistically significant.

## Additional Information

**How to cite this article**: Pandzic, E. *et al*. The ATP binding cassette transporter, ABCG1, localizes to cortical actin filaments. *Sci. Rep.* **7**, 42025; doi: 10.1038/srep42025 (2017).

**Publisher's note:** Springer Nature remains neutral with regard to jurisdictional claims in published maps and institutional affiliations.

## Supplementary Material

Supplementary Figures
