# Supplementary material for: The ATP binding cassette transporter, ABCG1, localizes to cortical actin filaments
Source: Sci Rep. 2017 Feb 6;7:42025. doi: 10.1038/srep42025 (PMC5292732; doi:10.1038/srep42025)
Supplement: Supplementary Figures [file srep42025-s1.doc]

**The ATP binding cassette transporter, ABCG1, localizes to cortical actin filaments**Elvis Pandzic^1,2^, Ingrid C. Gelissen^3^, Renee Whan^1^, Philip J. Barter^4,5^, Dmitri Sviridov^6^, Katharina Gaus^2,7^, Kerry-Anne Rye^4,5^ and Blake J. Cochran^4,*^

^1^Biomedical Imaging Facility, Mark Wainwright Analytical Centre, UNSW Australia, Sydney, Australia
^2^EMBL Australia Node in Single Molecule Science, School of Medical Sciences, UNSW Australia, Sydney, Australia
^3^Faculty of Pharmacy, University of Sydney, Sydney, Australia
^4^School of Medical Sciences, Faculty of Medicine, UNSW Australia, Sydney, Australia
^5^Faculty of Medicine, University of Sydney, Australia
^6^Baker IDI Heart and Diabetes Institute, Melbourne, Australia
^7^ARC Centre of Excellence in Advanced Molecular Imaging, UNSW Australia, Sydney, Australia

*Corresponding author: Blake J. Cochran, School of Medical Sciences, Faculty of Medicine, UNSW Australia 2052. [b.cochran@unsw.edu.au](mailto:b.cochran@unsw.edu.au). +61414248618


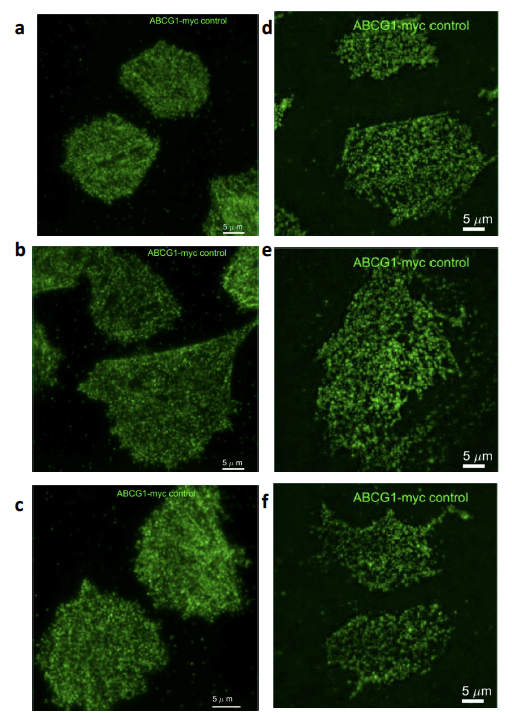


**Supplementary Figure 1:** Examples of control sample images of intact CHO-K1 cells (**a, b, c**) and cell membrane sheets (**d, e, f**) stained for ABCG1-myc.


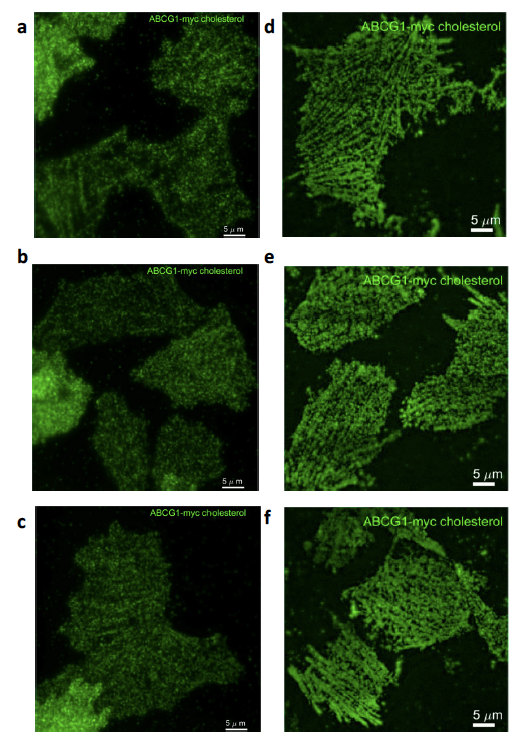


**Supplementary Figure 2:** Examples of cholesterol treated sample images of intact CHO-K1 cells (**a, b, c**) and cell membrane sheets (**d, e, f**) stained for ABCG1-myc.

**
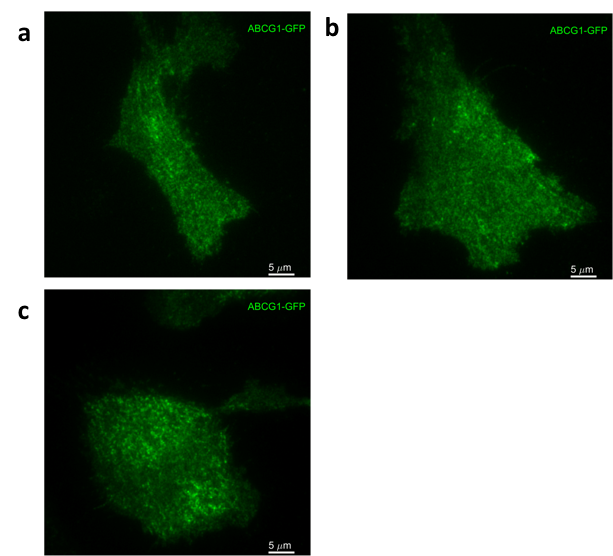
**

**Supplementary Figure 3:** Examples of control sample images of intact HeLa ABCG1-eGFP cells (**a, b, c**).

**
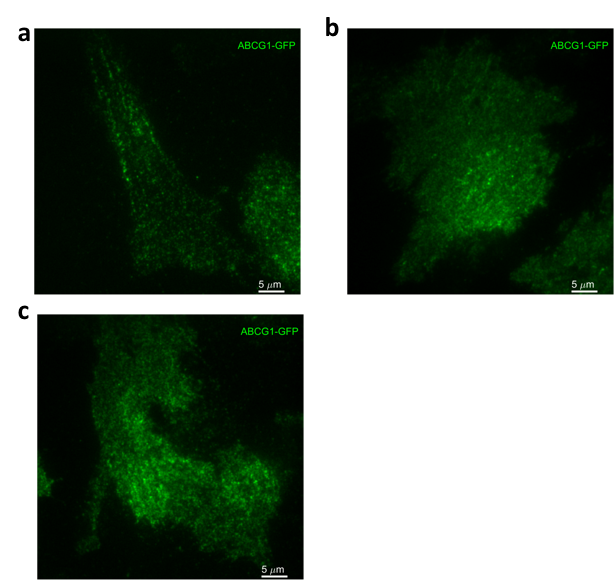
**

**Supplementary Figure 4:** Examples of cholesterol treated sample images of intact HeLa ABCG1-eGFP cells (**a, b, c**).


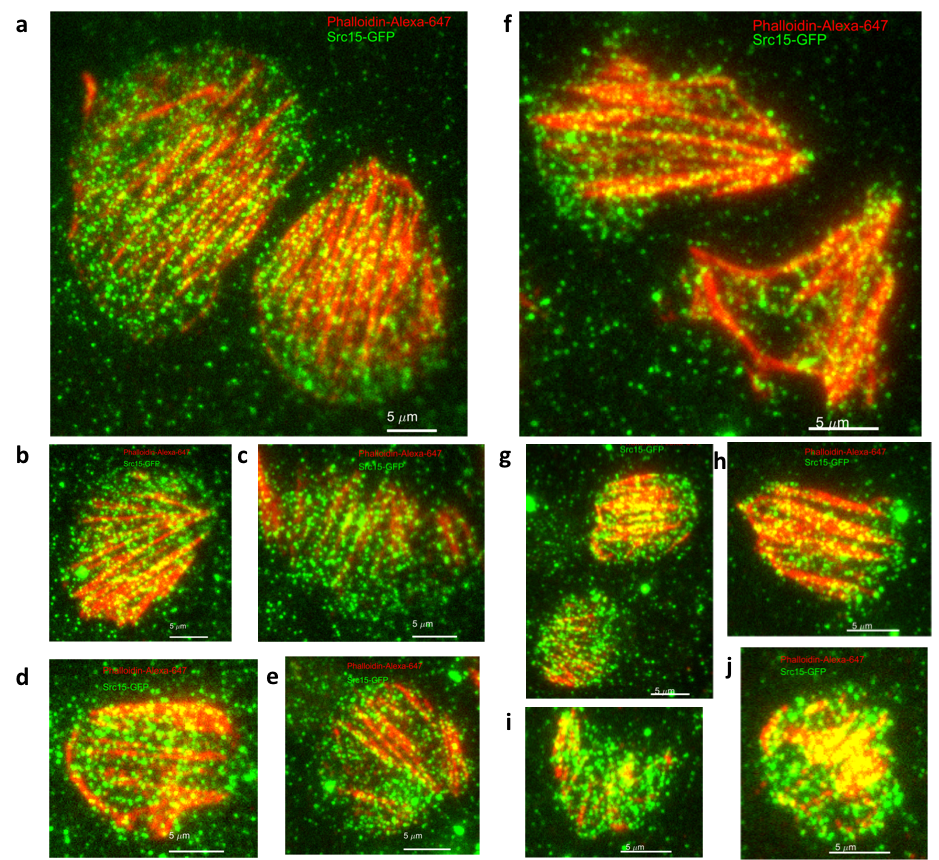


**Supplementary Figure 5:** **Cholesterol treatment does not alter Scr15 localisation.** Src15-eGFP (green) and actin (red) localisation in control (**a-e**) and cholesterol treated (**f-j**) cell membrane sheets.


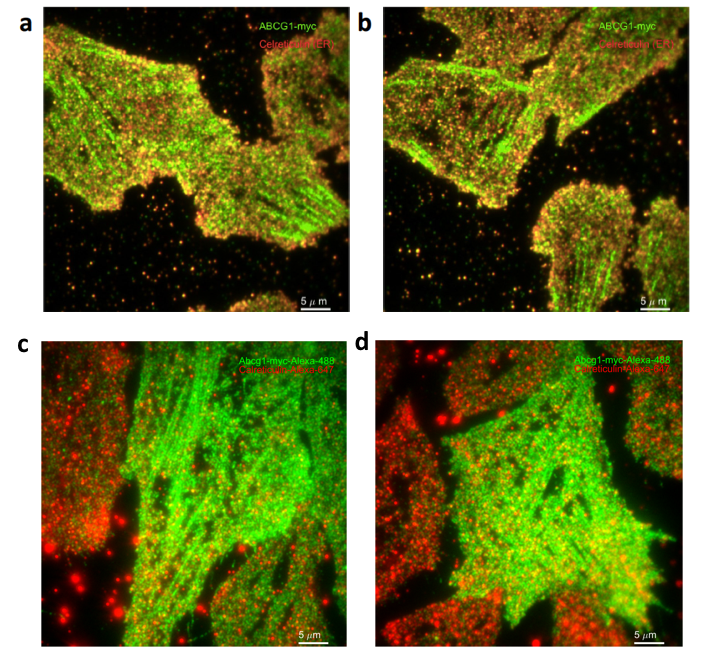


**Supplementary Figure 6:** Examples of control (**a, b**) and cholesterol treated (**c, d**) sample images of intact CHO-K1 cells stained for ABCG1-myc (green) and the endoplasmic reticulum marker calreticulin (red).


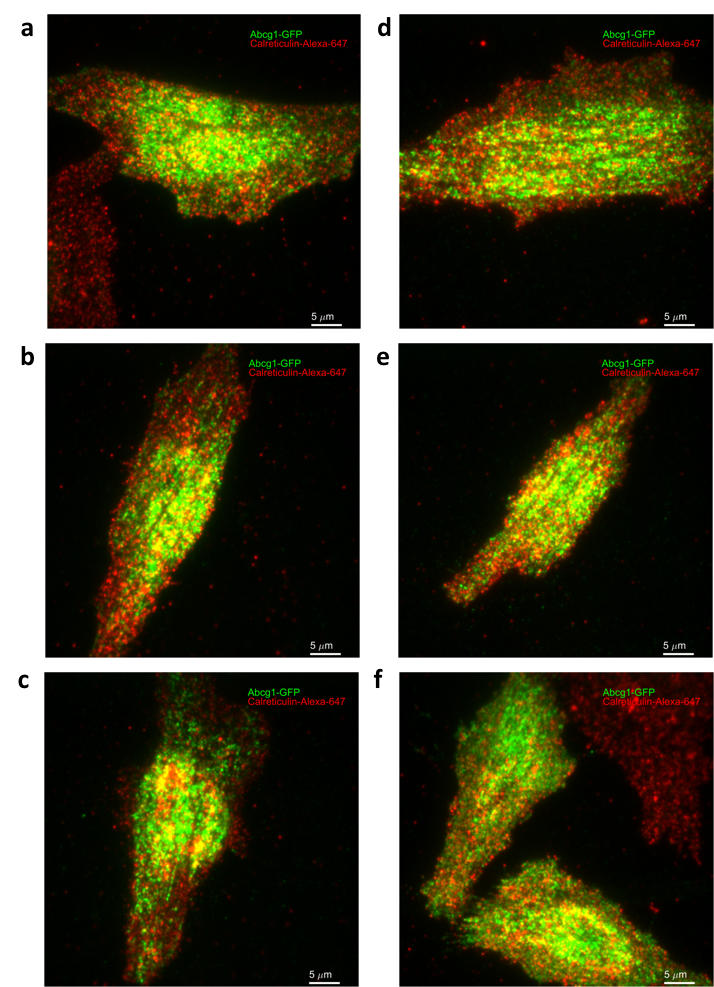


**Supplementary Figure 7:** Examples of control (**a, b, c**) and cholesterol treated (**d, e, f**) sample images of intact HeLa ABCG1-eGFP cells stained the endoplasmic reticulum marker calreticulin (red).


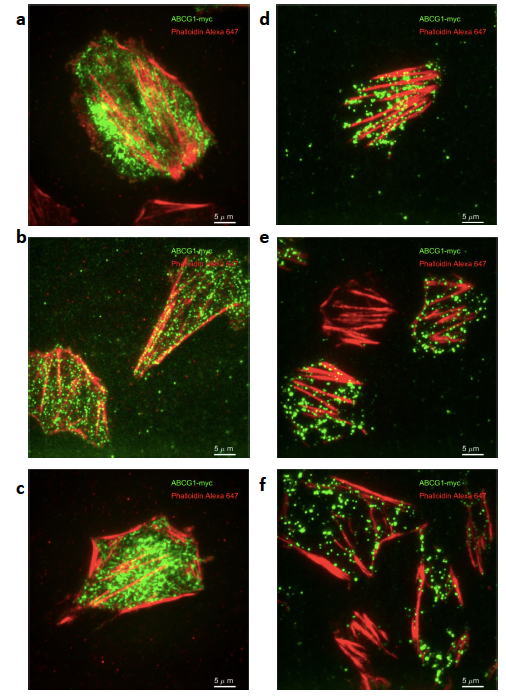


**Supplementary Figure 8:** Examples of control sample images of intact CHO-K1 cells (**a, b, c**) and cell membrane sheets (**d, e, f**) stained for ABCG1-myc (green) and actin (red).


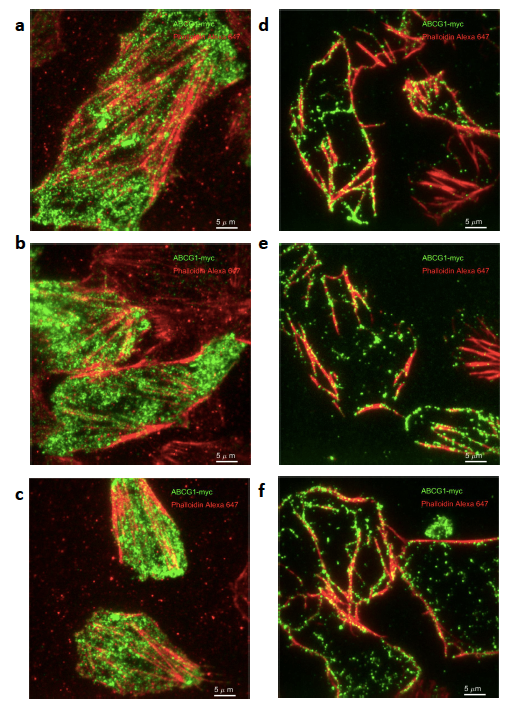


**Supplementary Figure 9:** Examples of cholesterol treated sample images of intact CHO-K1 cells (**a, b, c**) and cell membrane sheets (**d, e, f**) stained for ABCG1-myc (green) and actin (red).


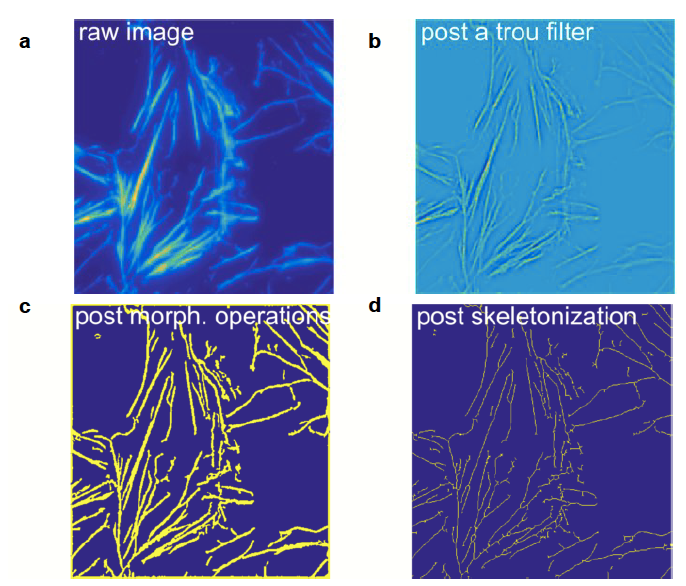


**Supplementary Figure 10:** Actin filament skeletonization procedure. (**a**) Raw image. (**b**) Original image filtered using *a trou wavelet* filter. (**c**) Morphological operations are applied to a thresholded image in order to obtain a cleaned binary mark of the actin network. (**d**) Skeletonization operator is applied to the cleaned mark in order to obtain the bare skeleton on actin filaments.


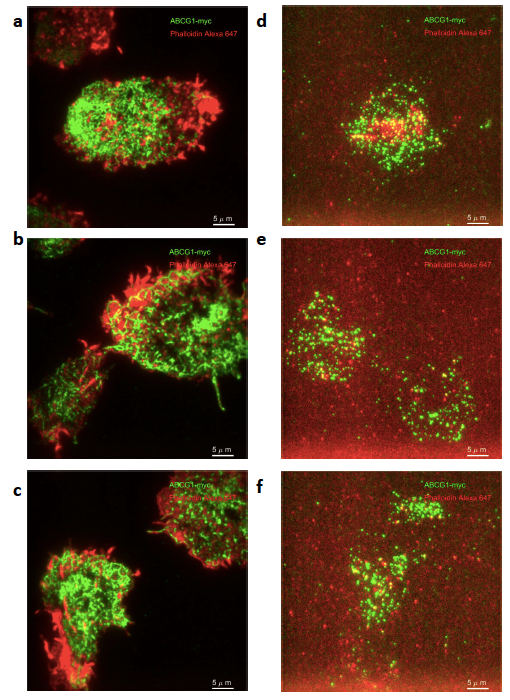


**Supplementary Figure 11**: Examples of Latrunculin D treated sample images of intact cells (**a, b, c**) and cell membrane sheets (**d, e, f**) stained for ABCG1-myc (green) and actin (red).
